# Color Tunable Composite Phosphor Ceramics Based on SrAlSiN_3_:Eu^2+^/Lu_3_Al_5_O_12_:Ce^3+^ for High-Power and High-Color-Rendering-Index White LEDs/LDs Lighting

**DOI:** 10.3390/ma16176007

**Published:** 2023-08-31

**Authors:** Shenrui Ye, Yukun Li, Ming Qiang, Wenhui Lou, Bo Dai, Hui Lin, Zhaoxia Han, Ruijin Hong, Dawei Zhang

**Affiliations:** 1 Engineering Research Center of Optical Instrument and System, Ministry of Education and Shanghai Key Laboratory of Modern Optical System, University of Shanghai for Science and Technology, No. 516 Jungong Road, Shanghai 200093, China; yeshenrui2021@163.com (S.Y.); louwenhui2023@163.com (W.L.); lioneldai2014@163.com (B.D.); hanzhaoxia0810@163.com (Z.H.); rjhongcn@163.com (R.H.); dwzhang@usst.edu.cn (D.Z.); 2 Ningbo Institute of Materials Technology and Engineering, Chinese Academy of Sciences, 1219 West Zhongguan Road, Ningbo 315201, China; suxyl3@nottingham.edu.cn; 3 Department of Chemical and Environmental Engineering, University of Nottingham Ningbo China, 199 Taikang East Road, Ningbo 315201, China; 4 Shanghai Institute of Optics and Fine Mechanics, Chinese Academy of Sciences, Laboratory of Micro-Nano Optoelectronic Materials and Devices, Key Laboratory of Materials for High-Power Laser, Shanghai 201800, China; 15955331164@163.com

**Keywords:** low-temperature sintering, bi-layer composite phosphor, SrAlSiN_3_:Eu^2+^-PiG, Lu_3_Al_5_O_12_:Ce phosphor ceramics

## Abstract

Lu_3_Al_5_O_12_:Ce^3+^ phosphor ceramics were fabricated by vacuum sintering. On this basis, a bi-layer composite phosphor was prepared by low-temperature sintering to cover the phosphor ceramics with a layer of SrAlSiN_3_:Eu^2+^-phosphor-in-glass (PiG). The optical, thermal, and colorimetric properties of LuAG:Ce^3+^ phosphor ceramics, SrAlSiN_3_:Eu^2+^ phosphors and SrAlSiN_3_:Eu^2+^-PiG were studied individually. Combining the bi-layer composite phosphors with the blue LED chip, it is found that the spectrum can be adjusted by varying the doping concentration of SrAlSiN_3_:Eu^2+^-PiG and the thickness of Lu_3_Al_5_O_12_:Ce^3+^ phosphor ceramics. The maximal color rendering index value of the white LED is 86, and the R9 is 61. Under the excitation of a laser diode, the maximum phosphor conversion efficacy of the bi-layer composite phosphors is 120 lm/W, the R_a_ is 83, and the correlated color temperature is 4534 K. These results show that the bi-layer composite phosphor ceramic is a candidate material to achieve high color rendering index for high brightness lighting.

## 1. Introduction

Since entering the 21st century, as the concept of green and sustainable development has been paid more and more attention, white LEDs (wLEDs) have been widely used in the field of lighting in recent years due to their long working life, low-energy consumption, positive effects on the environment, reliability, and safety, and have become the fourth generation of solid-state lighting sources after incandescent lamps, phosphor lamps, and gas discharge lamps [1,2,3]. In order to meet the needs of vehicle lighting, road lighting, medical lighting, marine lighting and so on, lighting systems based on wLEDs are developing in the direction of high power/high brightness (HP/HB) and high color rendering index (CRI). Currently, there are two main ways to prepare wLEDs: one is to use ultraviolet (UV) chips to excite red, green, and blue phosphors, for which broadband emission, high CRI, and tunable correlated color temperature (CCT) can be expected. However, currently, UV LED chips, especially those of high power, are expensive, and the leakage of UV light needs to be avoided [4]. In addition, the excitation and emission of different-type phosphors often overlap and cause re-absorption of the luminescence as a result, the luminous efficacy of the light source will be affected. The other main way to obtain LED-based white light is to utilize the blue LED chip to stimulate the yellow phosphor. This is the mainstream technology of the current commercial wLEDs for lighting applications. Specifically, the blue LED chip excites YAG:Ce^3+^ yellow phosphor, and the yellowgreen light emitted by the chip mixes with the blue light of the chip itself to produce white light, which is called phosphor-converted (PC) wLED [5,6,7,8]. For PC wLEDs, the Y_3_Al_5_O_12_:Ce^3+^ (YAG:Ce^3+^) phosphor powders are usually encapsulated in silicone gel however, due to the low thermal conductivity and the unsatisfying thermal stability of the silicone gel, this type of PC wLED are not suitable for HP/HB lighting [9,10,11,12].

To overcome the above problems, the researchers have developed three all-inorganic phosphors, such as single crystal (SC) [13,14], phosphor-in-glass (PiG) [15,16,17], and transparent ceramic (TC) [18,19,20]. Compared with phosphors and single crystals, phosphor transparent ceramics, which have high thermal and mechanical properties, an adjustable microstructure, higher light extraction efficiency, and small degradation of luminous efficacy and spectral shift, are generally considered to be a very promising and competitive candidate [21,22,23]. YAG:Ce phosphor ceramics have been extensively studied in recent years, showing high light conversion efficiency and thermal stability, but their low CRI and high CCT make them unsuitable for high-quality lighting applications [24]. To solve the problems, researchers have improved CRI by co-doping red emitting ions (Cr^3+^, Pr^3+^, Sm^3+^) [25,26,27,28] or by partially replacing Y^3+^ with different ions (Gd^3+^, Tb^3+^, Mg^2+^-Si^4+^) [29,30,31] to induce the emission redshift of Ce^3+^ in YAG, but this usually leads to a decrease in the luminous efficacy.

Lu_3_Al_5_O_12_:Ce^3+^ (LuAG:Ce^3+^) is a color converter with high luminous efficacy, reliability, thermal stability, and thermal conductivity (4~6 W·m^−1^·K^−1^), and strong green light emission peaked at about 507 nm [32,33,34,35,36]. Green light, as another important component in white light, plays an important role in LED lighting or displays. However, due to the serious lack of red light components in the spectrum of LuAG:Ce^3+^, it cannot be used alone in wLED lighting. In order to solve this problem, Cai et al. doped the red light emitting ion Mn^2+^ in LuAG:Ce^3+^, which made up for the red light component in LuAG:Ce^3+^ and achieved white light illumination with higher CRI (83). However, high concentrations of Mn^2+^ doping can lead to a sharp decrease in luminous efficacy [37]. Pricha et al. prepared bi-layer composite ceramics with CaAlSiN_3_:Eu^2+^ and YAG:Ce^3+^. The problem is that in the process of temperature heat treatment, serious chemical reactions occur at the interface of the two compounds, and the formed secondary phase restricts the performance of the phosphors, especially the nitride red phosphor [38]. In order to avoid interface reaction problems, Liu et al. prepared red emission (Sr,Ca)AlSiN_3_:Eu^2+^ and cyan emission BaSi_2_N_2_O_2_:Eu^2+^ phosphors into thin films and coated them on YAG:Ce^3+^ phosphor ceramics to prepare a full-spectrum color converter [39]. However, organic binders are used in the preparation of the film, making it unsuitable for HP/HB operation.

Low-temperature sintering of SrAlSiN_3_:Eu^2+^-PiG(Red-PiG) over LuAG:Ce^3+^ ceramics is a promising way to avoid degradation of the nitride red phosphors. Bao et al. reported the SrAlSiN_3_:Eu^2+^-PiG with low sintering temperatures, which exhibited excellent optical and chemical properties of borosilicate glass prepared with LTA and boron trioxide as the raw powders for the glass matrix of PiG, and which can also effectively prevent the chemical interaction between glass matrix and crystalline phosphor particles’ interface caused by the denaturation of SrAlSiN_3_:Eu^2+^(SASN:Eu^2+^) [40].

In this work, in order to achieve HP/HB and high CRI lighting, we use low-temperature sintering to overlay the red-emitting SrAlSiN_3_:Eu^2+^-PiG on the LuAG:Ce^3+^ phosphor ceramics. The effect of the thickness of the LuAG:Ce^3+^ phosphor ceramics and the concentration of the SrAlSiN_3_:Eu^2+^ in PiG on the optical, thermal, and colorimetric properties of bi-layer composite phosphors were studied. At the same time, the effect of different stacking structures of the bi-layer composite phosphors on the photoluminescence characteristics at specific concentrations was also studied. In addition, the optimal bi-layer phosphor is combined with the blue laser diode (LD) to assess its suitability for white lighting.

## 2. Experimental

### 2.1. Bi-Layer Composite Phosphor Was Prepared

Lu_2.991_Al_5_O_12_:Ce_0.009_ phosphor ceramics were prepared by high-temperature solid-state vacuum sintering. Commercial Lu_2_O_3_ (99.99%), α-Al_2_O_3_ (99.99%), and CeO_2_ (99.99%) were used as the raw materials, and 1 wt% PEG-400 (99.99%), 0.1 wt% MgO (99.99%), and 0.5 wt% tetraethyl orthosilicate (TEOS, 99.99%) were added as the dispersant and sintering additives. Using anhydrous ethanol as the grinding medium, the ball was ground for 16 h at the rotating speed of 250 r/min, and dried in the oven at 120 °C. The dried powders were screened by 100 mesh, and after 5 h of thermal treatment in air at 800 °C to remove organic ingredients, they were formed by uniaxial pressing in a stainless-steel die and then cold isostatic pressing under a pressure of 250 MPa. The obtained green bodies were pre-sintered in a vacuum at 1780 °C for 5 h and annealed at 1400 °C for 5 h. After that, the prepared phosphor ceramics were cut and polished into 10 × 10 mm squares (thickness: T = 0.5, 1 and 1.5 mm) for subsequent characterizations and preparation of bi-layer phosphor ceramics.

The commercial red phosphor SrAlSiN_3_:Eu^2+^ (SASN:Eu^2+^) powders were then mixed with the borosilicate glass powder to produce Red-PiG. In total, 68 wt% LTA zeolite (Na_12_Al_12_Si_12_O_48_·27H_2_O) and 32 wt% B_2_O_3_ (98%) were weighed and then loaded into a ball milling tank and mixed evenly. After the powders were heated to 1200 °C and held for an hour, the flowing melt was poured into the water for quenching, and Na_2_O-Al_2_O_3_-SiO_2_-B_2_O_3_ transparent glass was obtained. Then, the glass chips are thoroughly ground and sieved (100 mesh), mixing the SASN:Eu^2+^ phosphor powders with the glass powders with different weight ratios to obtain Xwt% SrAlSiN_3_:Eu^2+^ phosphor glass powder (X = 10, 20, and 30).

First, the mixed powders consisting of the glass matrix and the Xwt% SrAlSiN_3_:Eu^2+^ phosphor were evenly spread on the surface of LuAG:Ce^3+^ phosphor ceramics of different thicknesses; then together loaded into a muffle furnace at 800 °C to conduct direct thermal treatment for 15 min; and finally taken out from the muffle furnace. In this way, the bi-layer phosphor ceramic samples were obtained. The bi-layer phosphor ceramic samples are noted as “Xwt% SrAlSiN_3_:Eu^2+^-PiG/T mm LuAG:Ce^3+^” (Xwt% is the doping concentration of red phosphor in PiGs, X = 10, 20, and 30 wt%, T is the thickness of LuAG:Ce phosphor ceramics, and T = 0.5, 1, and 1.5 mm).

### 2.2. Bi-Layer Composite Phosphor of Characterizations

The crystal structure of LuAG:Ce^3+^ phosphor ceramics, Red-PiG, and bi-layer composite phosphors was tested with an X-ray diffractometer in a scanning step size of 0.01° in a range of 2θ from 10 to 80° (Rigaku SmartLab, Akishima City, Tokyo, Japan). The room temperature photoluminescence (PL), photoluminescence excitation (PLE) spectra, and temperature-dependent PL spectra of the samples were measured with a fluorescence spectrophotometer (Edinburgh Instruments, FLS1000, Edinburgh, UK) in the temperature range of 25 °C to 175 °C. The prepared bi-layer composite phosphor is mounted onto a GaInN-based blue LED chip to demonstrate the wLED prototypes. The luminescence spectra, luminous flux, phosphor conversion efficacy, CCT, and CRI of wLEDs were tested in a photometric, colorimetric, and electrical parameters test system (Everfine, ATA-500, Hangzhou City, China). The photometric and colorimetric properties of the laser-driven bi-layer composite phosphor in a photometric, colorimetric, and electrical parameters test system, including phosphor conversion efficacy and color coordinates, CCT, and CRI.

## 3. Discussion

**Figure 1:**
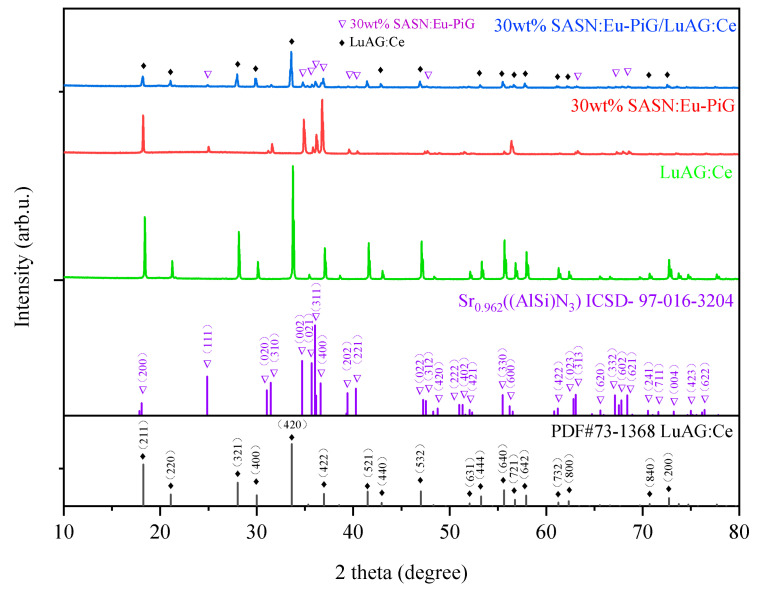
XRD θ–2θ scanning patterns of the LuAG:Ce^3+^ phosphor ceramic, the 30 wt% SASN:Eu^2+^-PiG, and the 30 wt% SASN:Eu^2+^-PiG/0.5 mm LuAG:Ce bi-layer composite phosphor.

Figure 1 shows the LuAG:Ce^3+^ phosphor ceramic, 30 wt% SASN:Eu^2+^-PiG, and 30 wt% SASN:Eu^2+^-PiG/0.5 mm LuAG:Ce^3+^ bi-layer composite phosphor of the XRD θ–2θ scanning pattern. The XRD patterns of 30 wt% SASN:Eu^2+^-PiG/0.5 mm LuAG:Ce^3+^ bi-layer composite phosphor show the characteristic peaks of LuAG:Ce^3+^ and SASN:Eu^2+^-PiG. The absence of secondary phases indicates that LuAG:Ce^3+^ phosphor ceramic in the bi-layer composite phosphor maintains a single-phase structure, and there are no impurities in SASN:Eu^2+^-PiG. The XRD peak intensity of the 30 wt% SASN:Eu^2+^-PiG/0.5 mm LuAG:Ce^3+^ bi-layer composite phosphor is drastically decreased because of the following reason: since the LuAG:Ce^3+^ phosphor ceramic is covered with a layer of 30 wt% SASN:Eu^2+^-PiG, so the diffraction of LuAG:Ce^3+^ phosphor ceramics is weakened due to the height of the LuAG:Ce^3+^ surface (lower than the optimal height for the diffraction detection) and the blocking by the SASN:Eu^2+^-PiG layer. The results showed that no severe chemical reaction occurred at the interface between LuAG:Ce^3+^ phosphor ceramic and SASN:Eu^2+^-PiG at a sintering temperature of 800 °C for 15 min.

**Figure 2:**
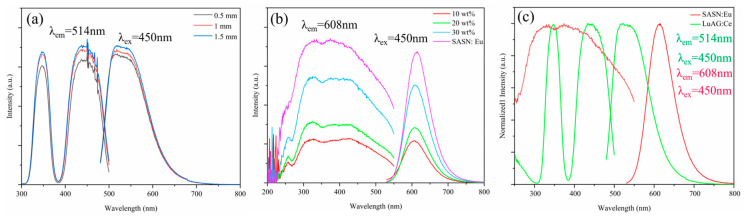
PL and PLE spectra of the (**a**) T mm LuAG:Ce^3+^ phosphor ceramic (T = 0.5, 1 and 1.5); (**b**) Xwt% SASN:Eu^2+^-PiG (X = 10, 20 and 30) and SASN:Eu^2+^ phosphor; and (**c**) normalized PL and PLE spectra of the LuAG:Ce^3+^ phosphor ceramics and SASN:Eu^2+^ phosphors.

Figure 2 shows LuAG:Ce^3+^ phosphor ceramics of different thicknesses, SASN:Eu^2+^ phosphors, and Xwt% SASN:Eu^2+^-PiG (x = 10 wt%, 20 wt%, and 30 wt%) of PLE and PL spectra. From Figure 2a, at 350 nm and 450 nm, LuAG:Ce^3+^ has two absorption bands, which are attributed to the 4f→5d^2^ and 4f→5d^1^ transitions of Ce^3+^, respectively [41,42]. The emission at 520 nm is due to the transition of Ce^3+^ from the 5d excited state to the ground states of ^2^F_5/2_ and ^2^F_7/2_. It can also be observed from Figure 2a that the emission intensity of LuAG:Ce^3+^ increases with the thickness of the LuAG:Ce^3+^ phosphor ceramic. From Figure 2b, SASN:Eu^2+^ exhibits broadband red emission centered at 608 nm due to the energy level transition of 4f^6^5d^1^→4f^7^ of Eu^2+^ under 450 nm excitation [43]. It is worth noting that LuAG:Ce^3+^ phosphor ceramics and SASN:Eu^2+^ phosphors can be excited simultaneously by the 450 nm blue light. Combined with the PL spectra of Figure 2c, the PL spectra of SASN:Eu^2+^ phosphors and LuAG:Ce^3+^ phosphor ceramics can cover the yellowgreen and red parts, which can compensate for the lack of red light when LuAG:Ce^3+^ phosphor ceramics are used alone. However, there is a large overlap between the PLE spectrum of SASN:Eu^2+^ and the PL spectrum of LuAG:Ce^3+^, which indicates that the yellowgreen light emitted by LuAG:Ce^3+^ phosphor ceramics can be absorbed by SASN:Eu^2+^-PiG. Therefore, in the preparation of white LED by bi-layer composite phosphors, Red-PiG is usually placed between the excitation light source (GaInN-based blue LED chip) and LuAG:Ce^3+^ phosphor ceramics to reduce the absorption of yellow and green light by Red-PiG.

**Figure 3:**
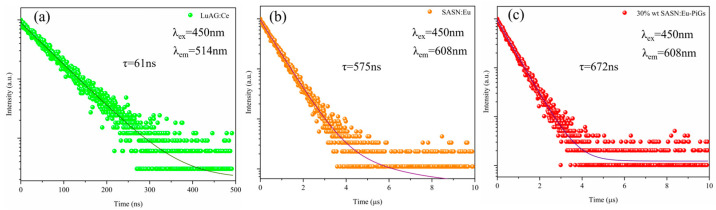
PL decay curves for (**a**) LuAG:Ce^3+^ ceramic, (**b**) SASN:Eu^2+^, and (**c**) SASN:Eu^2+^-PiG.

Figure 3 shows the PL decay curves for the LuAG:Ce^3+^ phosphor ceramic at 520 nm and for SASN:Eu^2+^ and 30 wt% SASN:Eu^2+^-PiG at 608 nm at room temperatur. A second-order exponential decay can be used to fit the curves, as shown below:

(1) $$y=y_{0}+A_{1}e^{-(x-x_{0})/t_{1}}+A_{2}e^{-(x-x_{0})/t_{2}}$$

where *y* is the intensity of the emission at moment *x*; *A*_1_, *A*_2_, *x*_0_ and *y*_0_ are constants; *t*_1_ and *t*_2_ denote the fast decay time and the slow decay time of the exponential component. The average lifetime t* can be obtained from the following equation [44]:

(2) $$t^{*}={(A}_{1}t_{1}^{2}+A_{2}t_{2}^{2})/(A_{1}t_{1}+A_{2}t_{2})$$

As can be seen in Figure 3a, Ce^3+^ in LuAG phosphor ceramics shows a mono-exponential fluorescence decay model with a lifetime of approximately 61 ns. As shown in Figure 3b,c for SASN:Eu^2+^ and 30 wt% SASN:Eu^2+^-PiG, their fluorescence lifetimes are 575 ns and 672 ns, respectively. In YAG:Ce^3+^, the yellow emission of Ce^3+^ ions belongs to the 5d-4f electron transition, for which the fluorescence lifetime is short, usually in the range of several to tens of nanoseconds (here in our experiment, it is 61 ns). The red emission of SASN:Eu^2+^ also belongs to the 5d-4f electron transition, for which the fluorescence lifetime is a little longer than that of YAG:Ce^3+^, usually in the range of hundreds of nanoseconds to ~1 microseconds (here in our experiment, it is 575 ns). The measured fluorescence lifetime of SASN:Eu^2+^-PiG is longer than that of SASN:Eu^2+^ phosphor powders, which could be attributed to a slight interface reaction at the interface between the SASN:Eu^2+^ phosphor particles and the glass matrix, which may introduce defects at the phosphor particles’ surface and in result may affect the transition routes of the electrons at the excited state.

**Figure 4:**
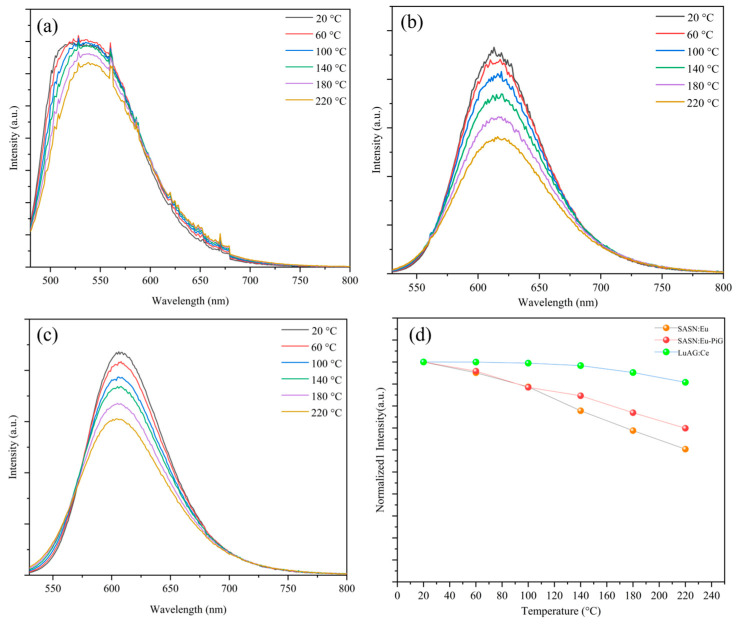
Temperature-dependent PL spectra of (**a**) LuAG:Ce^3+^ phosphor ceramic; (**b**) SASN:Eu^2+^ phosphor; (**c**) SASN:Eu^2+^-PiG; and (**d**) normalized temperature-dependent PL intensities of the three types of phosphor.

The thermal stability of the luminescence is another important criterion for judging whether bi-layer phosphors can be used in HP/HB lighting. As shown in Figure 4, in the range of 20–220 °C, the PL spectra of LuAG:Ce^3+^ phosphor ceramics, SASN:Eu^2+^ phosphor and 30 wt% SASN:Eu^2+^-PiG were tested at 450 nm excitation. With the increase in temperature, the PL intensity of all samples decreased. At 140 °C, the PL intensity of the LuAG:Ce^3+^ phosphor ceramics decreases to 98% at room temperature, maintaining good luminescence properties. The PL intensity of SASN:Eu^2+^ phosphor and 30 wt% SASN:Eu^2+^-PiG decreases to 77% and 84% of room temperature, respectively, at 140 °C, which is caused by the return of excited electrons to the ground state by the non-radiative transition. The above results show that the thermal stability of LuAG:Ce^3+^ phosphor ceramics and SASN:Eu^2+^-PiG is similar to that of single crystal LuAG:Ce^3+^ (at 200 °C, the PL intensity of LuAG:Ce^3+^ single crystal is 103% of the PL intensity at room temperature) and SASN:Eu^2+^ (at 430 K, the PL intensity of SASN:Eu^2+^ single crystal is 80% of the PL intensity at room temperature) [45,46]. Moreover, with the increase in temperature, the emission peak undergoes a slight blue shift, which is caused by the thermal reverse transfer of electrons from the low-energy excited state to the high-energy excited state with the help of thermally activated phonons [47]. In previous studies, the temperature at which the emission intensity of a sample is reduced to 50% of the initial intensity is referred to as the quenching temperature (T_50%_), which is one of the key indicators for determining the thermal stability of a sample [48,49]. In this work, the T_50%_ of LuAG:Ce^3+^ phosphor ceramics, SASN:Eu^2+^ phosphor, and 30 wt% SASN:Eu^2+^-PiG all exceeded 200 °C.

It is well known that since the higher the activation value, the lower the probability of non-radiative transitions. To further verify the thermal quenching performance of composite phosphors, we plot the scatter plot of SASN:Eu phosphor and 30 wt% SASN:Eu^2+^-PiG PL intensity over temperature, and the activation energy can be calculated using the Arrhenius equation [50] as follows:

(3) $$I\left(T\right)=\frac{I_{0}}{1+Aexp\left(\frac{-∆E}{KT}\right)}$$

**Figure 5:**
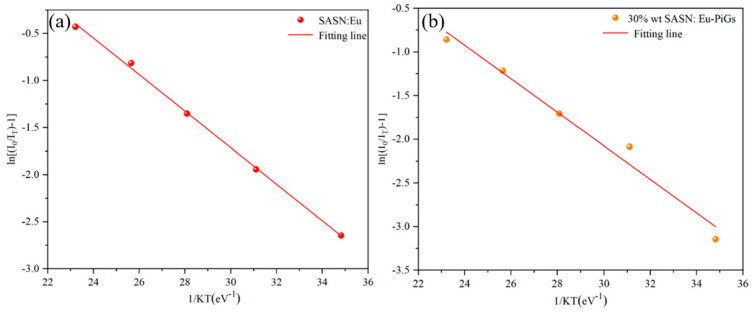
The thermal quenching activation energy of (**a**) SASN:Eu^2+^ and (**b**) 30 wt% SASN:Eu^2+^ − PiG.

*I*_0_ and *I*(*T*) are the PL intensities of the samples at room temperature and temperature *T*, respectively *A* is a constant, *K* is the Boltzmann constant (8.617 × 10^−5^ eV·K^−1^), and ∆*E* is the activation energy of thermal quenching. A scatter plot of the relationship between ln(*I*_0_/*I*(*T*)^−1^) and 1/*kT* was plotted based on the variable temperature spectra of SASN:Eu^2+^ phosphor, as well as 30 wt% SASN:Eu^2+^-PiG in the temperature range of 294–494 K, as shown in Figure 5, and from the slope of the fitted curves in the figure, SASN:Eu^2+^ phosphor and 30 wt% SASN:Eu^2+^-PiG with Δ*E* of 0.194 eV and 0.192 eV, respectively, the thermal activation energy did not show a significant decrease after preparing SASN:Eu^2+^ phosphor into PiG. The results showed that the Δ*E* of 30 wt% SASN:Eu^2+^-PiG was higher than that of most red phosphors, such as LiScGeO_4_:Cr^3+^ (0.067 eV) [51], La_2_MgZrO_6_:Cr^3+^ (0.089 eV) [52], and Y_2_O_3_:Eu^3+^ (0.17 eV) [53]. In summary, the two kinds of materials for the preparation of bi-layer composite phosphors, LuAG:Ce^3+^ and SASN:Eu^2+^-PiG, have good luminescence thermal stability.

**Figure 6:**
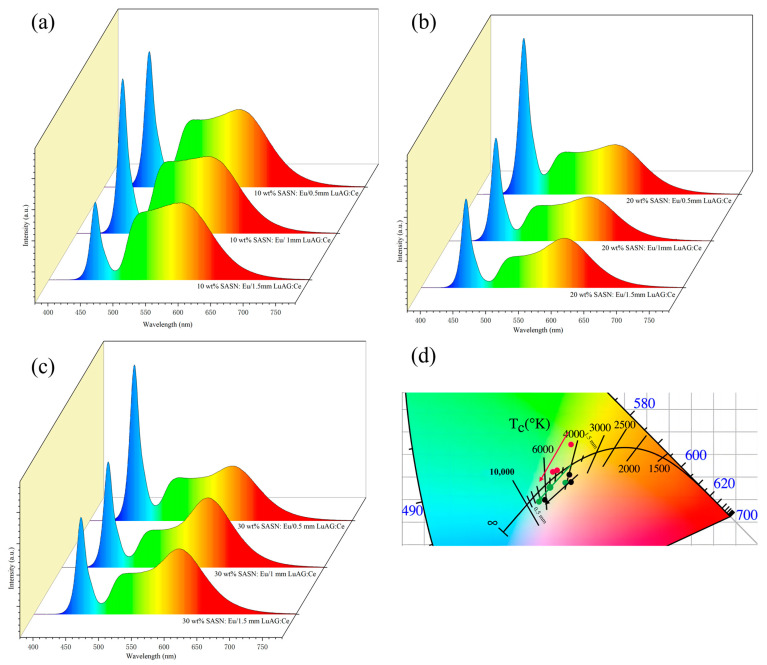
(**a**–**c**) EL spectra and (**d**) CIE chromaticity coordinates of Xwt% SASN:Eu^2+^-PiG/T mm LuAG:Ce^3+^ (X = 10, 20 and 30, T = 0.5, 1 and 1) bi-layer composite phosphors under excitation of 10 W of 450 nm blue LED input power.

In order to find the optimum spectral composition, as shown in Table 1 and Figure 6, the colorimetric properties of bi-layer composite phosphors with different concentrations of SASN:Eu^2+^-PiG and different thicknesses of LuAG:Ce^3+^ phosphors ceramics were tested under the excitation of a 450 nm blue LED chip. From Table 1 and Figure 6a–c, the CRI of bi-layer composite phosphors increases with the increase in SASN:Eu^2+^-PiG doping concentration in the composite phosphors, and when the doping concentration of SASN:Eu^2+^-PiG is 30%, its CRI can reach 86, while R9 also reaches 61. As with the addition of SASN:Eu^2+^-PiG, it is able to compensate for the missing red component in the spectrum of LuAG:Ce^3+^ phosphor ceramics. As shown in Figure 6d, due to the change in ceramic thickness, the proportions of red, yellow, and blue light in the emitted light are different. As the thickness of the LuAG:Ce^3+^ phosphor ceramic in the composite phosphor decreases, the CRI and CCT of wLED gradually increase, and the emitted light gradually changes from warm light to cold light. The thickness of the phosphor ceramic and the doping concentration of Red-PiG can therefore be purposefully adjusted to influence the CIE and CCT of bi-layer composite phosphors, thus enabling spectral modulation for neutral white lighting or the design of wLEDs for special lighting applications.

**Figure 7:**
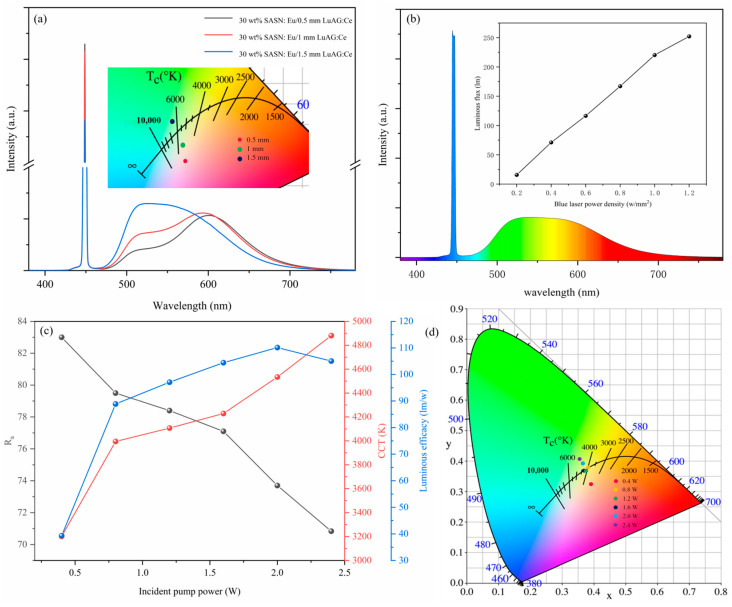
(**a**) Under the excitation of 1 W 450 nm blue laser, the luminescence spectra of bi-layer composite phosphors with 30 wt% Red-PiG/(0.5–1.5) mm LuAG:C; The inset shows the CIE chromaticity coordinates of a bi-layer composite phosphor; (**b**) emission spectra of 30 wt% Red-PiG/0.5 mm LuAG:Ce^3+^ bi-layer composite phosphors under the excitation of 1.2 W·mm^−2^ 450 nm blue laser. The inset shows correlation between luminous flux and blue laser power density; (**c**) CCT, phosphor conversion efficacy, and R_a_; and (**d**) CIE chromaticity coordinates of 30 wt% Red-PiG/0.5 mm LuAG:Ce^3+^ bi-layer composite phosphors at different incident powers.

In order to evaluate the application potential of bi-layer composite phosphors in laser-phosphor conversion illumination, the optical performance of 30 wt% SASN:Eu^2+^-PiG/(0.5–1.5 mm) LuAG:Ce^3+^ bi-layer composite phosphor under 450 nm laser excitation was measured. The R_a_, phosphor conversion efficacy, CIE, and CCT of composite phosphors with different thicknesses of phosphor ceramics are shown in Table 2, and it can be seen in combination with Figure 7a that under the excitation of 1 W 450 nm blue light laser, with the increase of the thickness of LuAG:Ce^3+^ phosphor ceramics, the red light component in the emission spectrum of the bi-layer composite phosphor is reduced, the green light component is increased, and the emitted light gradually changes from warm light to cold light. This conclusion is contrary to the above conclusion obtained by using blue LED chip as an excitation light source, mainly because the wLEDs lighting device prepared by using blue LED chip is of the transmissive geometry, when the phosphor ceramic with small thickness can transmit more blue light components, so as the thickness of the phosphor ceramic decreases, its CCT will gradually increase; In the laser illumination test, reflective illumination is used, when the thickness of phosphor ceramics will provide more green light components, so with the increase of phosphor ceramic thickness the CCT in laser illumination is gradually increased. When the bi-layer composite phosphor is 30 wt% SASN:Eu^2+^-PiG/0.5 mm LuAG:Ce^3+^, its CCT is 4349 K and R_a_ is 85.

The luminous flux, phosphor conversion efficacy, CCT, and CIE of the 30 wt% SASN:Eu^2+^-PiG/0.5 mm LuAG:Ce^3+^ composite phosphor at different incident pump powers were shown in Figure 7b–d. The illustration in Figure 7b shows a correlation between luminous flux and blue laser power density: the luminous flux increases from 15.7 lm to 252.1 lm as the power density increases from 0.2 W/mm^2^ to 1.2 W/mm^2^, and the raise rate of luminous flux decreases when the power density is greater than 1 W/mm^2^, and combined with the luminous efficiency of Figure 7c, it is also found that when the incident pump power reaches 2 W, the thermal quenching phenomenon becomes obvious. With the increase of incident pump power, its phosphor conversion efficacy decreases, the R_a_ decreases significantly, and CCT increases significantly. Combined with the CIE of Figure 7d, when the incident power is 0.8–1.6 W, its CIE just falls on the blackbody radiation curve, so it can be considered that the white light driven in this power range is already similar to the high-quality white light in nature.

## 4. Conclusions

LuAG:Ce^3+^ phosphor ceramics were prepared by high-temperature solid phase method, then bi-layer composite phosphors were prepared by low-temperature sintering technology to cover the phosphor ceramic with a layer of SrAlSiN_3_:Eu^2+^-PiG (Red-emitting). PL, PLE, temperature-dependent PL spectra and PL decay curves of LuAG:Ce^3+^ phosphor ceramics, SrAlSiN_3_:Eu^2+^ phosphors and different doping concentrations of SrAlSiN_3_:Eu^2+^-PiG were teste respectively. By observing the PL spectra of LuAG:Ce^3+^ and SrAlSiN_3_:Eu^2+^, it is found that the two can be combined to prepare a bi-layer composite phosphor that can achieve high CRI, and LuAG:Ce^3+^ phosphor ceramics and SrAlSiN_3_:Eu^2+^-PiG also show excellent thermal stability. In addition, we found that the CIE and the CCT can be adjusted by adjusting the doping concentration of SrAlSiN_3_:Eu^2+^-PiG, and adjusting the thickness of the LuAG:Ce^3+^ phosphor ceramics. Combining a 450 nm blue chip with a 30 wt% Red-PiG/0.5 mm LuAG:Ce^3+^ composite phosphor ceramic yielded wLEDs with a CRI of 86. Under the excitation of 450 nm blue laser, the CCT, R_a_ and phosphor conversion efficacy of white light were 4349 K, 85, and 110 lm/W, respectively. The above results show that SrAlSiN_3_:Eu^2+^-PiG/LuAG:Ce^3+^ bi-layer composite phosphor ceramics can be used as potential candidates for HP/HB and high CRI of white LED/LD lighting.

## Figures and Tables

**Table 1:** Under excitation of 10 W of 450 nm blue LED input power, wLEDs optical characteristics based on bi-layer composite phosphors of LuAG:Ce^3+^ phosphor ceramics of different thicknesses and SASN:Eu^2+^-PiG of different concentrations were tested.

Bi-Layer Composite Phosphor	CIE (x, y)	CCT (K)	CRI	R_a_	R9
10 wt% Red-PiG/1.5 mm LuAG:Ce^3+^	(0.3800, 0.4228)	4292	61	72	−44
10 wt% Red-PiG/1 mm LuAG:Ce^3+^	(0.3382, 0.3622)	5287	70	79	−17
10 wt% Red-PiG/0.5 mm LuAG:Ce^3+^	(0.3463, 0.3648)	4999	73	80	−10
20 wt% Red-PiG/1.5 mm LuAG:Ce^3+^	(0.3668, 0.3376)	4105	80	85	22
20 wt% Red-PiG/1 mm LuAG:Ce^3+^	(0.3344, 0.3279)	5396	82	87	27
20 wt% Red-PiG/0.5 mm LuAG:Ce^3+^	(0.3075, 0.2945)	7256	86	90	55
30 wt% Red-PiG/1.5 mm LuAG:Ce^3+^	(0.3774, 0.353)	3895	83	88	26
30 wt% Red-PiG/1 mm LuAG:Ce^3+^	(0.3806, 0.3393)	3664	85	88	32
30 wt% Red-PiG/0.5 mm LuAG:Ce^3+^	(0.3215, 0.2985)	6194	86	90	62

**Table 2:** Under the excitation of 1 W 450 nm blue laser, optical characteristics based on bi-layer composite phosphors of 30 wt% Red-PiG/R(0.5–1.5 mm) LuAG:Ce^3+^.

Bi-Layer Composite Phosphor	CIE (x, y)	CCT (K)	Phosphor Conversion Efficacy (lm/w)	R_a_
30 wt% Red-PiG/1.5 mm LuAG:Ce^3+^	(0.3440, 0.353)	6484	77	70
30 wt% Red-PiG/1 mm LuAG:Ce^3+^	(0.3373, 0.2995)	5182	61	84
30 wt% Red-PiG/0.5 mm LuAG:Ce^3+^	(0.3445, 0.2612)	4349	49	85

## Data Availability

The data of this paper are available on request from the corresponding author.
